# Perovskite-type catalytic materials for environmental applications

**DOI:** 10.1088/1468-6996/16/3/036002

**Published:** 2015-05-20

**Authors:** Nitin Labhasetwar, Govindachetty Saravanan, Suresh Kumar Megarajan, Nilesh Manwar, Rohini Khobragade, Pradeep Doggali, Fabien Grasset

**Affiliations:** 1 CSIR-National Environmental Engineering Research Institute (CSIR-NEERI), Nehru Marg, Nagpur-440 020, India; 2 Academy of Scientific and Innovative Research (AcSIR), CSIR-National Environmental Engineering Research Institute (CSIR-NEERI), Nehru Marg, Nagpur-440 020, India; 3 Key Laboratory of Biobased Materials, Qingdao Institute of Bioenergy and Bioprocess Technology, Chinese Academy of Sciences, No.189 Songling Road, 266101 Qingdao, People’s Republic of China; 4 Université de Rennes 1, UMR Institut des Science Chimiques de Rennes UR1-CNRS 6226, Campus de Beaulieu, CS74205, F-35042 Rennes, France; 5 CNRS, LINK, UMI 3629, National Institute of Material Science, 1-1 Namiki, 305-0044, Tsukuba, Japan

**Keywords:** perovskite, catalyst, emission control, diesel soot, environmental catalysis

## Abstract

Perovskites are mixed-metal oxides that are attracting much scientific and application interest owing to their low price, adaptability, and thermal stability, which often depend on bulk and surface characteristics. These materials have been extensively explored for their catalytic, electrical, magnetic, and optical properties. They are promising candidates for the photocatalytic splitting of water and have also been extensively studied for environmental catalysis applications. Oxygen and cation non-stoichiometry can be tailored in a large number of perovskite compositions to achieve the desired catalytic activity, including multifunctional catalytic properties. Despite the extensive uses, the commercial success for this class of perovskite-based catalytic materials has not been achieved for vehicle exhaust emission control or for many other environmental applications. With recent advances in synthesis techniques, including the preparation of supported perovskites, and increasing understanding of promoted substitute perovskite-type materials, there is a growing interest in applied studies of perovskite-type catalytic materials. We have studied a number of perovskites based on Co, Mn, Ru, and Fe and their substituted compositions for their catalytic activity in terms of diesel soot oxidation, three-way catalysis, N_2_O decomposition, low-temperature CO oxidation, oxidation of volatile organic compounds, etc. The enhanced catalytic activity of these materials is attributed mainly to their altered redox properties, the promotional effect of co-ions, and the increased exposure of catalytically active transition metals in certain preparations. The recent lowering of sulfur content in fuel and concerns over the cost and availability of precious metals are responsible for renewed interest in perovskite-type catalysts for environmental applications.

## 1. Introduction

Owing to their high thermal stability, excellent oxidation activity, and low price; perovskites have been explored for a variety of environment-related and energy-related applications, including automobile exhaust purification, fuel cells, N_2_O decomposition, and water–gas shift reactions [1–4]. In recent years, efforts have also been made to study the properties of perovskites in terms of reactions such as chemical looping combustion (CLC) and photocatalytic water splitting [5, 6]. In this paper, perovskite-type oxides appear as a family of potential compounds for the purpose of gaining insight into the relationships between solid-state and catalytic properties. These isomorphic solids (general formula: ABO_3_) are highly versatile due to the flexibility in the chemical composition of perovskites with a large number of cations that can fit into both the A and B positions within the same crystalline structure (i.e., practically all the metals are stable in the perovskite lattice). Another key feature is the availability of multi-component perovskites, which can be synthesized by partial substitution of cations at either A or B sites. Perovskite oxides containing a lanthanide element at position A and a transition metal at position B are used more frequently in heterogeneous catalysis, obviously exploiting the catalytic properties of transition metals [7–11]. Part of the importance of perovskite-type catalysts is that a number of transition metals show excellent catalytic activity for a variety of reactions due to their electronic structure. However, most transition metals have a tendency to oxidize easily at higher temperatures in the presence of oxygen, which is why the noble metals, due to their stability under such conditions, are currently being used as catalysts. Although metal oxides are stable under oxidative conditions and are also often used as catalysts, they possess rather limited adaptability due to their simple binary composition. In contrast, perovskites, with their flexible ABO_3_ composition and structure, offer immense possibilities in terms of adaptation to control their properties, and this is probably one of the most important reasons for the extensive studies of perovskites.

Perovskite-type oxides ideally have a cubical crystal structure, wherein cations with a large ionic radius have 12–fold coordination with oxygen atoms, occupying A sites, and cations with a smaller ionic radius have 6–fold coordination, occupying B-sites. A and O form cubic closest packing, and B is in the octahedral voids in the packing. In an ideal structure, where the atoms are simply bonding to one another, the B–O distance is equal to *a*/2 (*a* = length of unit cell), whereas the A–O distance is equal to (*a*/√2) and the following relation between the ionic radii holds:

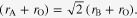


It has been found, however, that the cubic structure of perovskites can be still retained in an ABO_3_ structure even though this equation is not strictly obeyed. As a measure of this deviation from ideal cubical structure, Goldschmidt [12] introduced a *tolerance factor* (*t*), which is used to define the range of the ionic radii of A and B defined by the following equation, which is applicable at room temperature:

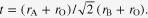


For an ideal perovskite, *t* is unity; however, the perovskite structure is also found for lower values of *t* in the range 0.75 to 1. In such cases, the cubical structure can be distorted into tetragonal, rhombohedral, or other lower symmetries [12]. It should be noted that perovskites with values of *t* greater than 1 having a hexagonal structure are also reported due to the larger ionic radii of the A ions or the smaller ionic radii of the B ions. BaNiO_3_ is a well-known example of a hexagonal perovskite structure where the value of *t* is 1.13 [13, 14].

### 1.1. Synthesis of perovskites

The physical properties of perovskites are greatly dependent on synthesis methods. In general, perovskites are usually formed at elevated temperatures, and the solid-state synthesis method is quite commonly used to prepare perovskites in pure form due to the availability of impurity-free precursors. Such synthesis methods are often suitable for electronic and electrical applications, but they pose problems when perovskites are subjected to surface-related studies. Obviously, perovskites with higher surface areas are required for catalytic and adsorption-related applications. Therefore, efforts have been devoted to synthesizing perovskites at lower temperatures with improved porosity. Close contact with precursors is essential for attaining the lowest possible synthesis temperature to achieve high surface area. The concept of maintaining high homogeneity of precursors (mixed-metal form) has been exploited for the purpose of achieving close contact with a view to synthesizing perovskites in the pure phase at low temperature. Low-temperature synthesis of perovskites results in higher surface area with smaller particle size, typically between the submicron and nano levels. Co-precipitation, citrate sol–gel, and solution combustion techniques have been commonly reported as ways to synthesize perovskites for catalytic applications. Serious attempts have been made to evaluate perovskite-based catalysts on a prototype scale, and the synthesis of perovskites on commercial honeycomb substrates has been successfully demonstrated. Some of the synthesis methods commonly used are briefly discussed here.

#### 1.1.1. Co-precipitation method

Co-precipitation is a useful technique for the synthesis of perovskites. In this technique, two or more components are precipitated by using a suitable precipitant to maintain a high degree of homogeneity of the precursors. Junwu *et al* synthesized nanocrystals of LaCoO_3_ using the co-precipitation method. In this method, La(NO_3_)_3_ and Co(NO_3_)_2_ were used as precursors and NaOH solution was used as a precipitating agent. After precipitation, the resulting product was centrifuged, washed with water and ethanol several times, dried at 100 °C, and calcined at 600 °C [15]. Muneeswaran *et al* synthesized nano-sized BiFeO_3_ powder by the co-precipitation method. Bi(NO_3_)_3_ · 5H_2_O and Fe(NO_3_)_3_ · 9H_2_O were dissolved in 200 mL of distilled water and stirred for 20 min to form a clear solution. The precursor solutions were precipitated using ammonia (2.5 M) at various pH levels, and it was found that the pure phase was observed at approximately pH 10 [16]. We have used the co-precipitation method extensively for the synthesis of several perovskite compositions, such as BaRuO_3_, La_3.5_Ru_4.0_O_13_, LaMnO_3_, La_0.8_Ba_0.2_MnO_3_, PrMnO_3_, Pr_0.8_Ba_0.2_MnO_3_, SrCoO_3_, and Sr_0.8_Ce_0.2_CoO_3_. It was observed that the pure crystalline phase of perovskites can be obtained by this method, but it results in a relatively moderate surface area [17–21]. Several precipitating agents have been reported based on the selection of A-site and B-site ions. We have observed that aqueous ammonia is one of the best precipitants, mainly due to its easy removal upon heating. It should be noted, however, that ammonia forms a complex with several metal ions and therefore it is not suitable as a precipitant in such cases. Co-precipitation is also used for preparing perovskites on ceramic structured supports, where homogeneous precipitate precursors can be easily coated onto a support for achieving a uniform perovskite on the support.

#### 1.1.2. Citrate sol–gel method

The sol–gel method is another useful technique for preparing perovskites with a relatively high surface area. In this method, the ‘sol’ gradually leads to the formation of a gel-like diphasic system, which has both a liquid and a solid phase. Decomposition of dried gel precursor materials through calcination yields the desired perovskites. Shabbir *et al* [22] reported a novel sol–gel process for preparing nano-sized perovskite-type LaFeO_3_ powder by thermal decomposition of the gel complex of LaFe–(C_6_H_8_O_7_ · H_2_O). The authors observed that optimization of the gelling conditions results in formation of the LaFeO_3_ perovskite phase without an explosion/combustion process and pH control. X-ray diffraction (XRD) results indicate that although the impurity phase (La_2_O_3_) appeared at ∼350 °C, the pure perovskite phase was observed above 600 °C with a particle size of approximately 25 nm. Guo *et al* [23] synthesized a series of dually substituted perovskite catalysts, La_1−*x*_K_*x*_Co_1−*y*_Pd_*y*_O_3−*δ*_ (*x* = 0, 0.1; *y* = 0, 0.05) by a citrate-based sol–gel process. A 1:1 molar ratio of citric acid to total metal ions was used. In this process, the formed solution after the addition of citric acid and metal salts was transferred into a vacuum rotary evaporator to remove the excess water at 60 °C. The viscous gel thus formed was dried homogeneously in air flow at 120 °C overnight to obtain a dried precursor. After pre-calcination at 350 °C for 2 h, the precursor was further calcined at 700 °C. We have explored the synthesis of LaCoO_3_ and La_0.9_Ba_0.1_CoO_3_ perovskites using this method. It was observed that although the surface area of the substituted catalyst La_0.9_Ba_0.1_CoO_3_ is slightly inferior, the material shows excellent catalytic activity for CO oxidation as well for particulate matter oxidation. In a recent study, we synthesized substituted PrMnO_3_ with various cations (K^+1^, Ce^+4^, Ba^+2^) at the A site using the sol–gel method. The surface area of perovskites was observed to be strongly dependent on the nature of the substituting element [24, 25].

#### 1.1.3. Solution combustion synthesis (SCS)

The combustion synthesis technique takes advantage of rapid, self-sustaining exothermic chemical reactions between a metal salt and a suitable organic fuel. Since most of the heat required for the synthesis is supplied by the reaction itself, the mixture of the reactants only needs heating up to a temperature that is significantly lower than the actual phase formation temperature. In recent years, this technique has been frequently adopted to produce very fine homogeneous crystalline powder, without decomposition of the intermediates and/or calcination steps, whereas these steps are rather necessary in conventional synthesis routes. This technique is particularly suited for producing nano-size particles of catalysts. A nano-structured catalyst coating over a trap can effectively improve local catalyst–soot contact conditions [26, 27]. The important condition that prevails in this synthesis is the generation of high localized temperatures for a very short time that nevertheless is long enough to cross the activation energy barrier for the formation of the perovskite phase. The evolution of a huge amount of gases results in the formation of a very foamy mass with smaller particles and a rough surface, which is often desired for catalytic applications. So far, little has been discussed about the gas emissions released during such synthesis methods and their suitability for the large-scale production of perovskites. This needs to be addressed before the technique can be commercially used for any number of perovskite compositions.

Russo *et al* synthesized a series of perovskite catalysts (LaMnO_3_, LaCrO_3_, LaFeO_3_, and LaNiO_3_) using the SCS method based on the exothermic and self-supporting reactions occurring between an aqueous solution of metal nitrates (acting as oxidizers) and urea (acting as a sacrificial fuel). The resultant perovskite crystals were between 45 and 75 nm in size with a high Brunauer–Emmett–Teller (BET) specific surface area of 15–20 m^2^ g^−1^ [28]. Fino *et al* used combustion synthesis to prepare LaMnO_3_, LaFeO_3_, LaCrO_3_, LaCr_0.9_O_3−*δ*_, and La_0.9_K_0.1_Cr_0.9_O_3−*δ*_ catalysts, which have a relatively high specific surface area of 18–25 m^2^ g^−1^. The authors explained that the synthesis can be split into two steps. The first is endothermic reaction, which represents the perovskite synthesis beginning with the metal nitrate precursors. The second reaction is exothermic and involves the reaction between oxygen derived from nitrate decomposition and urea [29]. Nanocrystalline Th-based perovskites (BaThO_3_ and SrThO_3_) were prepared through combustion synthesis by varying the fuel-to-oxidant ratio [30]. The nitrate salts of the precursors were dissolved with requisite amounts of water, and the aqueous solution of citrate with the desired stoichiometry was added. The resultant gel was calcined to get Th-based perovskites. The perovskites of iron, cobalt, and cerium (Sr_0.85_Ce_0.15_FeO_3*−x*_. La_0.6_Sr_0.4_Co_0.95_Fe_0.05_O_3*−x*_, BaCe_0.9_Y_0.1_O_3*−x*_.) were prepared through the auto-combustion method by varying the ratio of citrate and nitrate between 1 and 4. The morphology and the structural properties of these perovskites were influenced by the ratio of fuel and oxidant. No segregation of dopant was observed by maintaining a low fuel-to-oxidant ratio in this interesting study [31]. A number of Ce^3+^-based (CeCrO_3_, CeScO_3_) [32–34] as well as substituted Ce^3+^–based perovskites (La_1−*x*_Ce_*x*_CrO_3_, 0 ≤ *x* ≤ 1.0) [35] were prepared using the two-step synthesis method involving combustion followed by heating under a vacuum condition.

### 1.2. Supported perovskites

With a view to improving the surface area of perovskites, efforts have been made to synthesize perovskites directly on supports such as alumina and honeycomb supports. Among the reported methods, the supporting of perovskites as a thin layer or nanoparticles on an appropriate high-surface-area support is a potential method for practical applications [36]. The main problem with this method is a solid-state reaction between the precursors of the perovskite and the support at high temperature. Such synthesis methods, however, are also inevitable if perovskites as catalytic centers need to be dispersed onto supports for practical applications.

#### 1.2.1. Alumina-supported perovskite catalysts

We have been exploring the synthesis of various supported perovskites using both commonly available *γ*-alumina and ceramic cordierite honeycombs. In our previous study, we tried to synthesize LaRuO_3_ perovskite directly on *γ*-alumina through impregnation of a La^3+^ and Ru^3+^ mixed-metal solution [37]. Heating of these composites resulted in the formation of different undesirable phases, including aluminates. The perovskite phase of LaRuO_3_ could not be detected by XRD analysis. Therefore, lanthanum pre-coated and post-heated *γ*-alumina was used for the synthesis of LaRuO_3_ on *γ*-alumina that was first calcined at 950 °C for 10 h. The obtained powder was subsequently impregnated with La^3+^ salt solution and was heated at 700 °C for 3 h. This process was repeated several times. This alumina powder pre-coated with lanthanum oxide was then impregnated repeatedly with a mixed-metal ion solution of La^3+^ and Ru^3+^, followed by oven drying at 60 °C. A total 20 wt.% loading of LaRuO_3_ on pre-coated *γ*-alumina was targeted, and the concentration of precursor solution and impregnation cycles was optimized. The powder with La^3+^ and Ru^3+^ incorporated was subsequently heated at 850 °C for 12 h in a furnace. This resulted in *γ*-alumina-supported LaRuO_3_; however, a small number of other phases were also formed along with the LaRuO_3_ phase, as discussed elsewhere [37].

#### 1.2.2. Ceramic honeycomb–supported perovskite catalysts

As mentioned earlier, we have systematically studied the synthesis of perovskite catalyst–coated ceramic foam filters to assess the feasibility of the developed catalysts in realistic conditions. In a recent study from our group [38], we chose ceramic foam filters (CFF; 38 mm diameter, 50 mm length) made of *α*-Al_2_O_3_ for the preparation of perovskite-based catalyst-coated ceramic foam filters. This study was aimed at the synthesis of Pr_0.8_Sr_0.2_K_0.1_MnO_3_ on CFF. The catalyst was coated onto the CFF by the dip coating method. In this procedure, a CFF was soaked in the aqueous solution containing appropriate amounts of Pr(NO_3_)_2_ · 6H_2_O, Mn(CH_3_COO)_2_ · 4H_2_O, KNO_3_, and Sr(NO_3_)_2_ · 2H_2_O. The extra solution remaining in the pores of the ceramic foam filter was blown off by hot air, followed by drying and pre-calcination at 350 °C in air. This procedure was repeated three times, and the ceramic foam filter was calcined at 800 °C for 10 h. The perovskite catalyst coating observed in this method was approximately 3.12 wt%.

Some of the catalytic reactions of various perovskites for environment-related and energy-related applications are discussed here.

### 1.3. Perovskite-type catalysts for diesel soot oxidation

**Figure 1.**
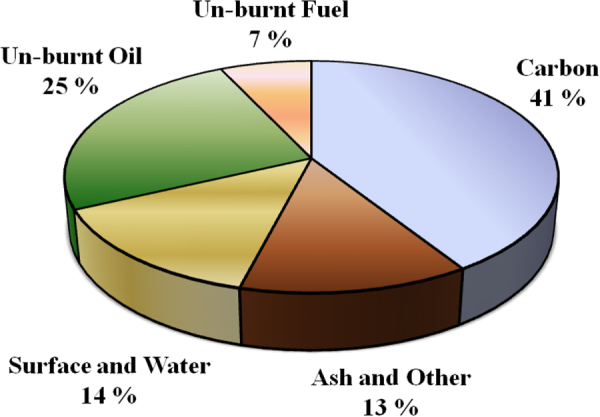
Particle matter composition of a heavy-duty diesel engine tested in a transient cycle as reported in [41]. Reproduced from D B Kittelson 1998 *J. Aerosol Sci.* **29** 575–88, with permission from Elsevier.

Diesel engines are the heart of transportation. Higher fuel economy, low maintenance requirements (especially in new-generation diesel engines), high durability, and low operating cost are the main advantages of diesel engines over gasoline engines. Diesel engines contribute, however, extensively to environmental pollution by emitting particulate matter (PM) and nitrogen-oxide (NO_*x*_) emissions. Soot has been identified as a prominent contributor to serious health and environmental issues [39, 40]. Diesel particulate matter is composed of carbon with small amounts of organic compounds as well as inorganic materials (ash and sulfates) as a result of unburned fuel, lubricating oil, etc. Typical diesel soot is composed of approximately 41% carbon, 25% unburned oil, 27% sulfate, approximately 13% ash, and 7% unburned fuel, as shown in figure 1 [41]; however, this composition can vary significantly depending on fuel composition and engine conditions.

PM emitted from diesel vehicles consists mainly of ultrafine particles, which can easily be inhaled inside the body and reach deep into lungs, causing serious health issues. PM is also responsible for a number of direct and indirect environmental impacts, including global warming (through the black carbon effect) and photochemical reactions leading to poor visibility [42]. Control of diesel particulate matter emissions has therefore become necessary, and stringent norms for PM emissions have been stipulated for new diesel vehicles. Several techniques are promising for the reduction of emissions from diesel engines. Modified and alternative fuels have been studied in an attempt to correlate emission levels with certain fuel specifications in order to optimize fuel composition for lower PM emissions. A small but significant reduction in diesel-regulated emissions can be achieved by modifying diesel fuels but at the expense of fuel cost [43]. Some engine modifications have proved very effective in reducing diesel engine emission levels and have been implemented in recent years. Diesel particulate standards were first proposed in 1981, and it was generally believed that filter-based particulate removal systems would meet future standards [44]. As a consequence, the redesign of engines is currently one of the main strategies for meeting current emission standards. After-treatment techniques have been studied extensively for particulate removal from diesel exhaust gases. Options such as fuel additives, diesel oxidation catalysts, partial flow filters (PFFs), and diesel particulate filters (DPFs) have been explored as potentially successful alternatives for controlling emissions. The use of DPFs is one of the most promising approaches for reducing particulate matter (PM) from diesel vehicles; however, effective regeneration of DPFs is a major limitation to their feasibility. Catalytic regeneration has been suggested as the best approach, and intensive efforts are being made to develop suitable catalysts for this application [45].

Although almost all commercial regenerative-type DPF technologies are based on noble metal catalysts, a range of other catalytic materials have been studied for their activity in terms of soot oxidation. These include noble metal–promoted transition metal oxides, mixed oxides, other metals, perovskites, bi-functional materials, eutectic mixtures, etc [46–49]. Perovskites have been proposed as alternative potential materials for this application, due mainly to their thermal stability, excellent oxidation activity, and low price. Studies of perovskites have confirmed their potential as catalytic materials for the regeneration of diesel particulate filters using the exhaust temperature. Their chemical stability has been a big challenge because of the significant sulfur content of diesel fuel, but perovskite catalysts can show improved stability with steep lowering of sulfur content. Russo *et al* have reported that Li-substituted chromite-type perovskite catalysts exhibit the highest activity for soot oxidation as a consequence of a large amount of weakly chemisorbed O- species (*α*–oxygen) [50]. Teraoka *et al* have also reported that mixed-metal oxides (e.g., La–K–Mn–O system) demonstrate activity toward the simultaneous removal of NO_*x*_ and soot. Activity toward soot oxidation and NO_*x*_-reduction selectivity was strongly dependent on potassium content [51]. Fino and Specchia have prepared nano-structured PrCrO_3_ on CeO_2_ through combustion synthesis, which showed even better catalytic activity than Pt/Al_2_O_3_ when the catalyst was coated onto a monolith [52].

Zhang *et al* have prepared a series of macroporous catalysts of La_1−*x*_K_*x*_Co_1−*y*_Fe_*y*_O_3_ (*x* = 0, 0.1; *y* = 0, 0.1) by a combined method of organic ligation and solution combustion. They found that the size of the soot was smaller than the pore size of the catalyst; this allows the soot to enter into the pore of the catalyst, resulting in good contact between soot and catalyst. As a consequence, macroporous La_0.9_K_0.1_CoO_3_ perovskite showed better catalytic activity than nano-size catalysts. The activity of the catalysts was found to be comparable to that of supported Pt-based catalysts [53].

Peng *et al* have reported a highly efficient catalyst (La_0.8_K_0.2_Cu_0.05_Mn_0.95_O_3_) for simultaneous removal of NO_*x*_ and diesel soot. The catalytic properties of this catalyst were tested under simulated diesel engine exhaust using temperature programmed reduction (TPR) and it was found that this catalyst showed promise for the simultaneous removal of NO_*x*_ and diesel soot [54]. Ura *et al* have examined soot and NO_*x*_ reaction on a K^+^-substituted SrTiO_3_ catalyst. The authors demonstrated that partial substitution of strontium by K^+^ induces oxygen vacancies that promote soot oxidation. The most efficient substitution observed by the authors was at *x* = 0.2. It was observed from the results of Sr_0.8_K_0.2_TiO_3_ that the soot ignition temperature was 380^o^C in a gas flow of 10% O_2_ in Ar and 240 °C in a gas flow containing 1500 ppm of NO_2_ + 10% O_2_ in Ar. A similar test was performed with inert material for comparison, and the soot ignition temperature was observed to be 635 °C and 570 °C in a gas flow of 10% O_2_ in Ar and 1500 ppm of NO_2_ + 10% O_2_ in Ar, respectively [55].

Bialobok *et al* have studied SrTiO_3_ catalysts doped with alkali metals (Li, K, Cs). They prepared two types of SrTiO_3_-based perovskite catalysts; one was substituted in the A position of the structure (Sr_1-*x*_Me_*x*_TiO_3_, *x* = 0.05–0.2), and another was impregnated with the same amount of alkali metals. Results indicate that the substituted catalysts showed improved catalytic activity over that of the impregnated catalysts [56]. Cauda *et al* have investigated an innovative multi-functional catalyst (La_0.9_K_0.1_Cr_0.9_O_3−δ_ + 1 wt.% Pt) for diesel soot combustion with combined direct and indirect (NO–NO_2_–NO cycle) oxidation mechanisms. This catalyst was applied over a wall-flow SiC trap and tested according to a standard protocol on an engine bench and showed excellent catalytic activity [57].

Our group has also extensively examined the soot oxidation reaction using various types of perovskite materials. La-based perovskite-type catalysts (La_3.5_Ru_4.0_O_13_) showed very high catalytic activity for soot oxidation. We observed that this catalytic material possesses the required thermal stability in comparison with LaRuO_3_. This may be due to the presence of ruthenium at the stable higher oxidation state of Ru^4+^ in the perovskite structure. The catalyst showed soot oxidation activity through dissociative adsorption of oxygen on the surface of the catalyst. It should be noted, however, that most of the catalytic materials demonstrated their soot oxidation activity only in the presence of NO_*x*_ [58]. We examined the catalytic activity as a function of Ce substitution in a SrCoO_3_-type perovskite catalyst for diesel soot oxidation. Small amounts of substitution of cerium at the A site of SrCoO_3_ resulted in improved redox properties and consequently improved catalytic activity. The promotional effects of cerium substitution in SrCoO_3_ and the presence of a small amount of potassium ions seem to be responsible for the observed high soot oxidation activity [19].

In a recent study, we reported Ba-substituted LaCoO_3_-type perovskite, which showed much improved catalytic activity for CO oxidation, even below 200 °C, as well as particulate matter oxidation at 370 °C. Although no clear difference in x-ray photoelectron spectroscopy studies of LaCoO_3_ and La_0.9_Ba_0.1_CoO_3_ was observed, temperature programmed desorption (TPD) experiments support the existence of small amounts of *α*-oxygen in La_0.9_Ba_0.1_CoO_3_, which may be responsible for the enhanced catalytic activity [59]. Bench-scale examination of our group of Pr_0.7_Sr_0.2_K_0.1_MnO_3_ perovskite catalyst–coated ceramic foam filters indicated that *T*_*i*_ (the temperature at which soot combustion is initiated) decreased by 150 °C and *T*_*f*_ (temperature at which soot combustion completed) by 100 °C for direct soot oxidation using real diesel engine exhaust. This may be considered high catalytic activity under realistic conditions [60].

### 1.4. N_2_O decomposition on perovskite catalysts

Over the last few decades, nitrous oxide has been considered one of the pollutants most responsible for the destruction of ozone in the stratosphere as well as being a relatively strong greenhouse gas [61–65]. It has been recognized that the migration of nitrous oxide (N_2_O) with chlorofluorocarbons (CFCs) into the stratosphere contributes to ozone layer depletion [66]. N_2_O is also an absorber of infrared radiation and thus contributes to the greenhouse effect [67, 68]. The ambient level of nitrous oxide is 324 ppb, which recently has been constantly increasing. Control of N_2_O emissions has attracted much interest due to the global warming potential of N_2_O (AR5, IPCC 2013), which is 265 times larger than that of CO_2_ [69, 70]. About 18 million tons of N_2_O are released each year into the atmosphere from both anthropogenic and natural sources [71]. The nitric and adipic acid industries are the major anthropogenic sources of N_2_O emissions [63, 70–73]. Nitric acid is being increasingly used for fertilizer production and is manufactured through oxidation of ammonia. During this catalytic oxidation process, nitrous oxide is produced as a byproduct. N_2_O equivalent to approximately 15.3 million tons of CO_2_ was emitted from the nitric acid industry in 2012 [70]. Nylon production is also responsible for nitrous oxide emission; in nylon production, adipic acid is used as one of the major precursors. N_2_O is emitted as a byproduct of adipic acid production during the oxidation of a ketone–alcohol mixture with nitric acid. N_2_O emissions from the adipic acid industry were estimated to be the equivalent of 5.8 million tons of CO_2_ in 2012 [70]. Hence, reduction or decomposition of N_2_O to control its emissions from industries is one of the important research areas in environmental catalysis.

Although a few methods for controlling N_2_O emissions are available, direct catalytic decomposition is considered a simple potential method for N_2_O removal. Different types of catalysts have been studied for the decomposition of N_2_O, including noble metals [74–77], metal oxides [78–82], and metal or ion exchanged zeolites [83–87]. At low temperatures, noble metal–based catalysts show very good activity in terms of N_2_O decomposition. However, these noble metal–supported systems are expensive and unstable at high temperatures as well as in the presence of oxygen [75]. Most zeolite-based materials are also potential catalysts for the decomposition of N_2_O at low temperatures. The development of hydrothermally stable zeolite-based catalysts has significantly improved prospects relative to their natural instability in the presence of water vapor [88]. Usually for industrial applications, catalysts with better chemical and thermal stability as well as having a lower cost are preferred. Perovskites are promising catalysts because of their low-cost excellent thermal stability as well as the possibility of tailoring their properties for N_2_O reduction and decomposition reactions. It is possible to activate N_2_O molecules on a transition metal associated with a perovskite structure, which can lead to decomposition of N_2_O into its constituents (N_2_ and O_2_), the most desirable reaction in terms of avoiding the use of reducing agents and harmful byproducts. Because perovskites offer various supra-facial and intra-facial reaction pathways, this has generated considerable interest among researchers in exploring perovskites for N_2_O control, and a large number of studies are therefore reported in the literature.

**Figure 2.**
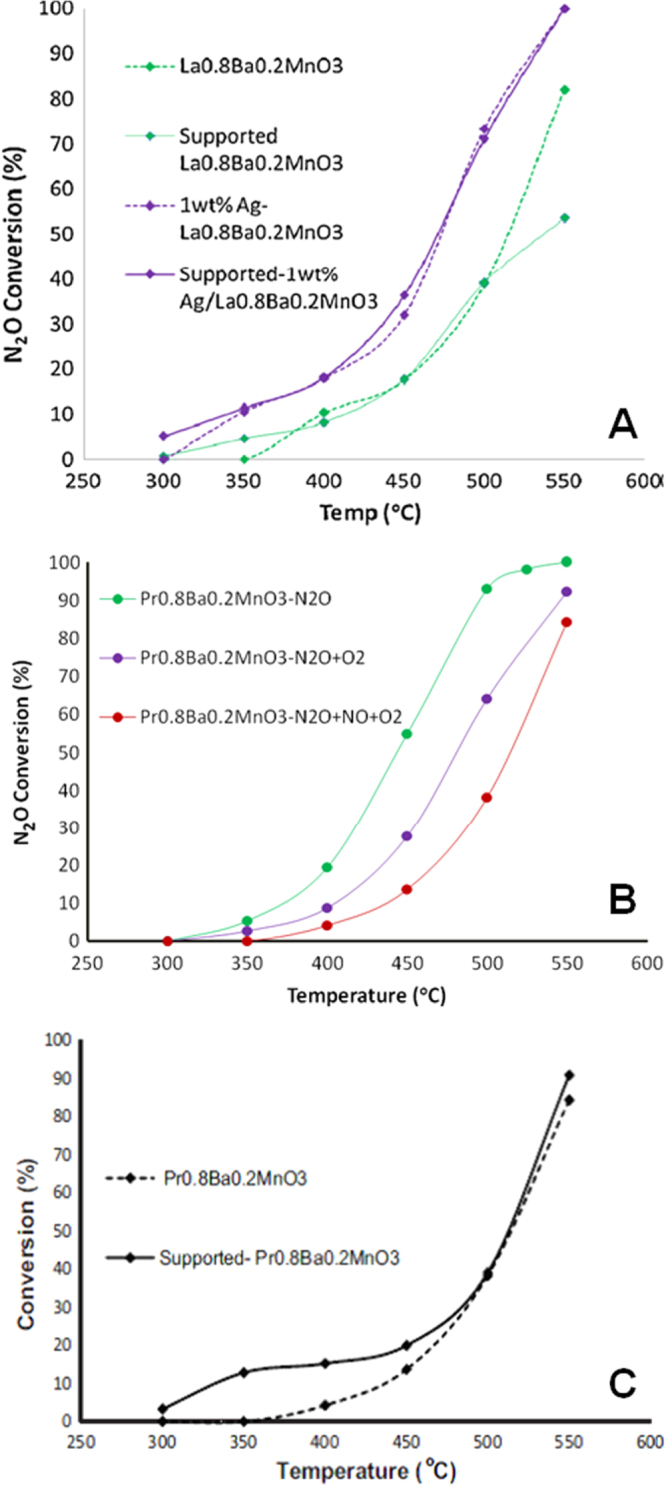
N_2_O decomposition as a function of temperature (0.5 vol.% N_2_O, 0.02 vol.% NO, and 5 vol.% O_2_): (a) La_0.8_Ba_0.2_MnO_3_, supported La_0.8_Ba_0.2_MnO_3_, 1 wt.% Ag/La_0.8_Ba_0.2_MnO_3_ and supported Ag (1 wt.%)/La_0.8_Ba_0.2_MnO_3_; (b) Pr_0.8_Ba_0.2_MnO_3_; and (c) supported Pr_0.8_Ba_0.2_MnO_3_ [93, 94]. Adapted from S Kumar *et al* 2011 *J. Mol. Catal. A: Chem.* **348** 42 and S Kumar *et al* 2012 *Catal. Today* **198** 125, with permission from Elsevier.

Russo *et al* have studied a LaCoO_3_ perovskite-type catalytic material prepared by solution combustion synthesis for N_2_O decomposition; they reported that 50% of N_2_O conversion can be achieved at 455 °C and 490 °C in the absence and presence of 5% oxygen, respectively [89]. Alini *et al* have studied a number of alkaline metal–based perovskite materials comprising Ca and Ba for decomposition of N_2_O. They reported that a CaMn_0.7_Cu_0.3_O_3_ catalyst showed the highest activity, 95% conversion, at 550 °C as well as stable catalytic activity for a simulated industrial gas coming from an adipic acid plant for a period of 1400 h [90]. This report has been considered a valuable finding, particularly regarding the stability of perovskites under an actual exhaust stream, which might be otherwise questioned due to the basic nature of many perovskite compositions, which are quite susceptible to attack by commonly present acidic gases like SO_2_. Dacquin *et al* have studied catalytic N_2_O decomposition on 1 wt.% Pd/LaCoO_3_. They reported a reaction rate of 0.0023 mmol min^−1^ g^−1^ at 460 °C [91]. Ivanov *et al* have studied the various compositions of a La–Sr–Fe–O perovskite catalyst for high-temperature N_2_O decomposition, and they have also discussed the oxygen mobility of perovskites for different reactions [92]. Recently our group also explored N_2_O decomposition using different AMnO_3_ (A = La and Pr) perovskite catalysts and reported the effect of partial substitution and Ag promotion on catalytic N_2_O decomposition using perovskites [93, 94]. It was observed that both Ba substitution and Ag incorporation showed a promotional effect in terms of N_2_O decomposition activity in a LaMnO_3_-type perovskite catalyst. A series of Ba-substituted as well as Ag-incorporated samples were evaluated for their activity in terms of N_2_O decomposition and the maximum/optimized activity observed for La_0.8_Ba_0.2_MnO_3_ compositions promoted with La_0.8_Ba_0.2_MnO_3_ (20 mol %) and Ag- (1 wt.%) (figure 2(a)). In the case of Pr-based perovskites, the maximum catalytic activity was observed for the Pr_0.8_Ba_0.2_MnO_3_ composition (figure 2(b)) [94]. O_2_ TPD and H_2_ TPR studies clearly inferred that the redox properties of Ba-substituted as well as Ag-promoted perovskites were altered, which correlates with their improved catalytic activity in terms of N_2_O decomposition. It is also important to study the N_2_O decomposition activity of supported catalysts since a number of physical properties, such as porosity and surface area, influence catalytic activity, and catalyst and support interactions can also alter catalytic activity. Hence, optimized compositions of La_0.8_Ba_0.2_MnO_3_, Pr_0.8_Ba_0.2_MnO_3_, and Ag (1 wt.%)/La_0.8_Ba_0.2_MnO_3_ were prepared in supported form using cordierite honeycomb support and evaluated for their catalytic activity. Interestingly, catalytic activity was observed for these supported catalyst samples that was nearly identical to that of the catalyst in powder form (figures 2(a) and (c)) [93, 94]. These results convincingly inferred the possibility of coating perovskites onto commercially available ceramic honeycomb substrates for practical applications.

### 1.5. Perovskites for chemical looping combustion

Power generation plays a pivotal role in economic growth and is one of the most influential factors, along with globalization, in the development of any nation [95]. Energy demand has increased substantially lately due to the accelerated increase in population, which is responsible for creating a gap between energy demand and supply. This demand, however, can be met by implementing high-capacity power generation plants [96]. Coal is one of the world’s most abundant and most widely distributed fossil fuels, and its use for power generation is continuously increasing worldwide. Of the total global electricity production, 44% is from coal, which is expected to remain a primary fuel source for power generation in the coming years to meet electricity demand, especially in developing countries [97]. Coal and methane are known as non-renewable fossil fuels and are associated with the release of an enormous amount of CO_2_ into the atmosphere through power generation activity [98]. It has been reported that fossil fuel–based thermal power plants emit about one-third of the total CO_2_ emissions, which are one of the major contributors to the greenhouse gas effect [98, 99]. It has been anticipated that installed fossil fuel-based thermal power plants around the world will most likely double by 2030 [100]. Therefore, it is essential to implement new CO_2_ capture and storage (CCS) strategies for the economical use of fossil fuels for power generation with mitigation of CO_2_ emissions. CCS is one of the potential options for mitigating CO_2_ emissions from various fossil fuel-based sources. CCS may reduce the amount of CO_2_ emitted into the atmosphere while allowing the continued use of fossil fuels for power generation at thermal power plants and other large-scale industries [101–103]. CCS technology has been successfully implemented in small-scale industries but not in large-scale industries, owing to the technical immaturity of the components of CCS technology as well as the requirement of more energy and the high cost [96]. The currently available CCS techniques capture about 85–95% of CO_2_; however, they require 10–40% additional energy for capturing and compressing CO_2_ from the large volume of flue gases [104].

**Figure 3.**
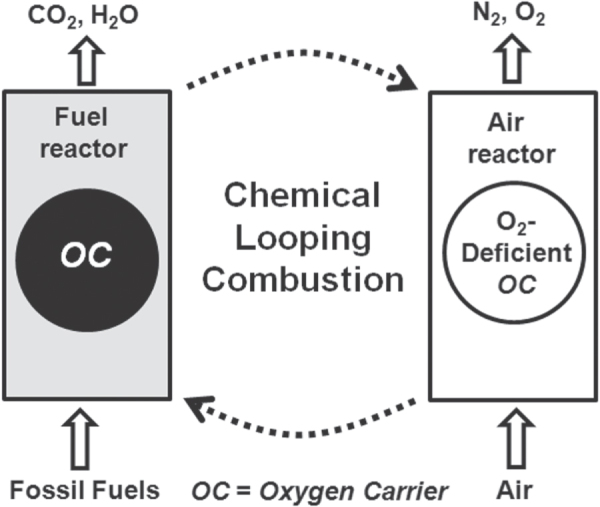
Schematic illustration of chemical looping combustion of an oxygen carrier.

CLC has the inherent potential of effecting CO_2_ separation from flue gases after the combustion reaction of fossil fuels, as shown in figure 3. This makes it one of the most efficient techniques for delivering sequestration-ready CO_2_ at lower cost. In the CLC process, oxygen can be derived from metal oxide referred to as an oxygen carrier [105–108]. The virtually pure CO_2_ formed in the CLC process can be readily captured and stored, or it can be directed as reactants to prepare value-added products [109, 110]. CLC possesses the potential for application in, for instance, clean energy generation or clean combustion, reforming, and hydrogen production [111–114]. Moreover, it has also been used for the combustion of coal with advanced techniques for coal gasification [115]. It has been reported that the transportation sector alone is responsible for about one-quarter of the total global CO_2_ emissions, and this can be expected to increase enormously in the coming years. A promising option is the use of H_2_ as a fuel to reduce CO_2_ emissions. However, the technologies for producing pure H_2_ are still under development and are also expensive. Therefore, H_2_ production from fossil-fuel sources (e.g., steam reforming of natural gas) is currently based on existing technologies, which are expected to continue to dominate in the coming years. Currently the production of H_2_ comes mostly from reforming natural gas (e.g., methane), partial oxidation of heavy oils and naphtha, and gasification of coal. Natural gas is a promising source for H_2_ production, where the ratio of H_2_ to carbon is roughly twice that of oil. The chemical looping reforming (CLR) process is a promising alternative to H_2_ production from natural gas. In CLR, a suitable oxygen carrier is circulated between two reactors as in CLC. In a fuel reactor, the fuel (e.g., CH_4_) is oxidized and forms syngas from the lattice oxygen of the oxygen carrier, whereas the reduced oxygen carrier after the removal of reactive oxygen is completely re-oxidized by air in the second reactor. In this way, the products are free from flue gases (e.g., N_2_) [116].

The practicability of the CLC and CLR processes strongly depends on the development of a suitable oxygen carrier, which is the key component in both chemical looping processes. Considerable efforts have been made in the last few decades regarding the development of oxygen carriers. A potential oxygen carrier should have the following characteristics: (i) abundant availability of raw materials to produce the oxygen carrier, (ii) non-toxicity, (iii) high oxygen carrying capacity, (iv) higher reactivity with fuels under harsh chemical looping conditions, (v) regeneration as well as attrition resistance properties, (vi) no carbon formation during chemical looping reactions, (vii) selectivity for product formation, (viii) low cost, and (ix) minimal environmental impacts. Individual transition metal oxides of Cu, Mn, Fe, Co, and Ni have been extensively investigated for chemical looping applications due to their favorable reductive/oxidative thermodynamics; however, single transition metal oxides pose a number of practical limitations as oxygen carriers due to their poor multi-cycle performance, carbon formation, agglomeration behavior under chemical looping conditions, etc [117–122]. Although individual transition oxides show inferior activity for long-term chemical looping applications, they have a number of advantages in terms of their physical and chemical properties and therefore should be exploited for their chemical looping applications, especially in mixed-oxide forms [122–131].

Perovskite-based mixed oxides with the general formula ABO_3_ have been widely exploited for catalysis applications—for instance, hydrogenation, CO oxidation, ammonia oxidation, and methane combustion. Perovskites have also been exploited recently in chemical looping applications owing to their desirable physiochemical properties—for instance, variable structural composition, excellent redox properties, high reactivity, high stability in a harsh thermal environment, high mobility of oxygen, and better mechanical properties. Perovskite-based mixed oxides have high oxygen mobility, which facilitates easy removal/insertion of oxygen from/into the structure [123–127].

La-based perovskite-type mixed oxides (LaBO_3_) have been widely exploited for chemical looping applications. LaFeO_3_ exhibits enhanced activity as well as selectivity for syngas formation in a fixed bed reactor, which makes it attractive as an oxygen carrier for chemical looping applications. The surface-adsorbed oxygen of LaFeO_3_ is highly active in completing the combustion of fuels, whereas the lattice oxygen is very selective in the formation of syngas (partial oxidation). It is well known that the substitution of cations (e.g., La^3+^) with a lower oxidation state (e.g., Sr) results in better chemical looping activity. La_*x*_Sr_1−*x*_FeO_3−*δ*_ has shown very high selectivity for syngas formation. The substitution of Sr in La-based perovskites increases the oxygen capacity of the materials but reduces the selectivity for syngas formation [116]. Partial substitution of Fe in Co sites of La_1−*x*_Sr_*x*_Co_*y*_Fe_1−*y*_O_3_ results in a slight decrease in structural stability; however, continuous oxygen supply has been observed in sequential reduction–oxidation cycles [123–127]. Furthermore, CO selectivity decreases dramatically with the increase in the amounts of Fe substitution and is not suitable for chemical looping reforming applications [128]. Nalbandian *et al* have studied La_1−*x*_Sr_*x*_M_*y*_Fe_1−*y*_O_3−*δ*_ synthesized by the citrate method as an oxygen carrier for CLR. The pure phase of La_0.7_Sr_0.3_FeO_3_ has shown moderate stability during multiple reduction–oxidation cycles. The addition of 5% NiO improved the yields of H_2_ as well as the stability of the perovskites. Among the La-based perovskites explored, La_0.7_Sr_0.3_Cr_0.05_Fe_0.95_O_3_ mixed with 5% NiO yielded up to 90% of H_2_ and also showed very good stability in multiple reduction–oxidation cycles. Thus, the addition of small quantities of NiO to La_0.7_Sr_0.3_Cr_0.05_Fe_0.95_O_3_ through mechanical mixing substantially increases methane decomposition as well as selectivity for the formation of syngas [116]. Fang *et al* have also studied La_1-*x*_Sr_*x*_FeO_3_ as oxygen carriers prepared by the combustion method for chemical looping reforming reactions of CH_4_. They demonstrated that Sr substitution in the range 0.3 to 0.5 resulted in better reactivity, selectivity, and oxygen-donation ability. They also concluded that substituted LaFeO_3_ shows promise as an oxygen carrier for CLR of methane [129]. Evdou *et al* have studied La_1−*x*_Sr_*x*_FeO_3−*δ*_ as redox materials for the simultaneous production of pure hydrogen and syngas in a membrane reactor. They concluded that the calcination temperature is an important parameter that influences chemical looping performance. The addition of small quantities of NiO to La_1−*x*_Sr_*x*_FeO_3_ substantially increases methane decomposition reactivity, selectivity for the formation of CO and H_2_, and water splitting activity [130]. Xiao *et al* have studied LaFeO_3_/Al_2_O_3_ prepared through a wet mixing–kneading method. They examined chemical looping reforming in a fixed bed reactor and a circulating fluidized bed reactor. The addition of Al_2_O_3_ to LaFeO_3_ improves oxygen migration for CH_4_ conversion, which forms syngas selectively. The reaction temperature and bed height of the fuel reactor greatly influence CH_4_ conversion and CO selectivity. The higher reactor temperature and bed height are shown to be beneficial for reforming applications with bubbling fluidized fuel reactors. LaFeO_3_ as an oxygen carrier showed potential in terms of CLR process, but it needs further modification in the manufacturing process to improve its structural stability and chemical properties to be used as a potential oxygen carrier [131].

### 1.6. Pervoskites in photocatalysis

In the historical background of photocatalysis [132], semiconductor materials have received exceptional attention in the cleaner energy arena due to increasing energy demand and the necessity of environmental remediation. With increasing global population, the world is facing energy challenges, and there is a pressing need for desirable and sustainable growth in the energy sector. In the absence of any attractive renewable sources of energy, the conventional ways of energy production are growing several-fold, with negative environmental impact. Therefore, developments related to effective photocatalytic strategies from harvesting solar energy to producing usable chemical fuel have received their share of attention. Photovoltaic cells have been a great success in this generation and are being put to use harvesting solar energy for direct conversion to electricity. This option, however, requires the storage of electric power generated through photovoltaic reactions. Perhaps this is one of the main reasons for exploring other photocatalytic options, which involve photocatalytic and photoelectrochemical (PEC) reactions as promising approaches to harnessing solar energy. The conversion of solar to chemical energy through water splitting reactions for the generation of hydrogen has therefore been explored for some time as an ideal reaction for sustainable energy generation.

Numerous potential applications of solar photocatalysis have been reported [133, 134]. Among these, energy and environmental issues were studied widely using a variety of photocatalysts. In general, an efficient photocatalyst, also called an ideal semiconductor, should include light harvesting and redox capabilities to facilitate the desired chemical reactions, thus achieving the targeted reaction. Inorganic semiconductor materials should have adequate capability to absorb solar energy across a broad spectrum. Because of their light harvesting property, potential photocatalysts absorb solar energy, leading to the generation of photoelectrons in the conduction band and holes in the valence band for their possible use in redox reactions. The redox nature of a photocatalyst as an intrinsic property dictates solar energy conversion efficiency. For this reason, TiO_2_ [135], SrTiO_3_ [136], and NaTaO_3_ [137] are among the most explored photocatalysts for hydrogen generation because they show the desirable stability, inert nature, and thermodynamically optimal band structures to realize photocatalytic reactions. Although TiO_2_-based photocatalysts show light harvesting properties and stability in the UV region, they cannot absorb visible light efficiently due to their large band gap (>3.2 eV). On the other hand, gallium arsenide can absorb light more effectively but is found to be too unstable as a photocatalytic system to be of practical value [138]. The continued progress driven by cleaner energy demand in this exciting research field has opened new avenues to the design and development of numerous photocatalyst compositions for a variety of applications ranging from wastewater treatment to water splitting and CO_2_ photoreduction. Researchers have also advanced the scientific aspects of semiconductor photocatalysis as well as those involving combinations of different mechanisms. Most semiconductors include metals/mixed metal oxides used as efficient photocatalysts (e.g., CdS [139, 140]). Furthermore, surface tuning of oxide nanomaterials can improve photocatalytic performance, as systematically reviewed by Jing *et al* [141]. Their efforts were mainly in the direction of the utilization of visible light in the solar spectrum. Mixed-oxide-type photocatalysts, including those of perovskite-type structures, are very prominent because of their broadly diverse properties. Recently ABO_3_-based photocatalysts were systematically reviewed with a view to their characteristic properties for water splitting reactions [142], which further confirm their potential role in photocatalysis.

We have explored a range of ABO_3_-type perovskite-based materials for sacrificial donor–assisted as well as overall water splitting reactions to generate hydrogen. With the exceptional adaptability of their properties through compositional variations, perovskites show promise for solar hydrogen production among the large number of photocatalysts being explored. They are also frequently explored in combination with other oxides to carry out various steps of complex uphill reactions involved in water splitting reactions. As already discussed, the flexible composition of perovskites is based on large-size rare earth or alkaline earth metals at the A site and transition metals at the B site in an ABO_3_-type structure. These perovskites can be tailored through band gap engineering approaches, and photocatalysts thus designed can harvest solar light more effectively. Perovskites thus synthesized show broadband absorption over the visible to near-infrared region of the solar spectrum. Another challenge related to the charge separation between photogenerated holes and electrons has also been undertaken with perovskite compositions to achieve overall improved efficiency [143].

We recently investigated metal ferrite-based photocatalysts (MFeO_3_, where M = La, Pr, and Ce) for water splitting reactions. LaFeO_3_ was synthesized in nanocrystalline form (crystallite size ∼38 nm), generating hydrogen through photocatalytic reaction [144]. This photocatalyst exhibited an optical band gap of 2.07 eV with an absorption spectrum predominantly in the visible region of the spectrum with a hydrogen evolution rate of 3315 *μ*mol g^−1^h^−1^ in the sacrificial donor–assisted water splitting reaction. Perovskites having Ce^3+^ ions play a key role in photocatalysis due to their allowed transitions by selection rules [145]. Their photocatalytic performance was improved significantly through decreasing the optical band gap value by increasing Ce^3+^ in the unit cells of perovskites, thus facilitating the absorption of light in the visible region [34]. Recently we also reported a PrFeO_3_-type perovskite composition [146] with a crystallite size of 20 nm, which shows a significant hydrogen evolution rate of 2847 *μ*mol g^−1^h^−1^. We have also synthesized CeFeO_3_ perovskite using different methods, showing a band gap of 2.1 eV, which suggests that the material can absorb light in the visible region. This material is being explored for its photocatalytic and PEC activity with regard to water splitting reactions. Initial PEC investigations of CeFeO_3_ show a significant photocurrent difference of approximately 0.6 mAcm^−2^ at 0.5 V between light and dark conditions. Detailed PEC evaluations of CeFeO_3_ and other perovskites are in progress. Most of our current efforts are being diverted toward achieving increased hydrogen yield, possibly using a visible spectrum of solar radiations.

**Figure 4.**
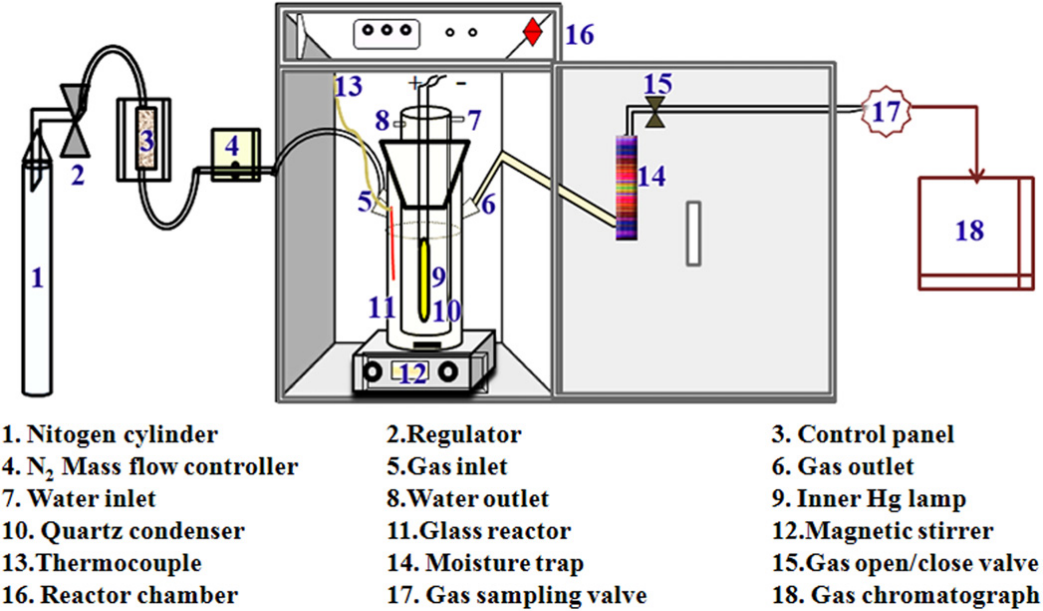
Schematic illustration of lab-scale evaluation systems for photocatalytic water splitting reactions.

In addition to the development of improved photocatalyst compositions, other considerations need attention when it comes to photocatalytic water splitting. Undeniably a major issue is photoreactor design as well as experimental setup and conditions that can significantly affect photocatalytic activity [147]. Various types of reactors are available for water splitting reactions, and a few of them are used in our studies. These include a 20 mL vial-type reactor for initial screening to optimize various parameters, including photocatalyst dose, sacrificial donors, and illumination intensity. After optimization of the parameters, photocatalytic water splitting reactions were further carried out in a tubular borosilicate/quartz photoreactor with an inlet port for inert gas purging and an outlet for automatic injection to a gas chromatograph (GC) or to collect the gas in a collector directly, as shown in figure 4. This setup uses typically about 200–400 mL of reaction mixture in a reactor of 750 mL volume with an efficient inner irradiation source. A high-pressure mercury lamp of 100–400 W (or any other source of illumination) is often used with a quartz inner jacket for photocatalysts with wide band gap when intensive UV light of wavelength shorter than about 300 nm is required. However, a Xe lamp with a cutoff filter is usually employed for visible light irradiation. A solar simulator that is a standard light source for evaluation of solar cells is also used in our case for simulating various wavelengths, including the so-called one-sun condition. A solar simulator with an air-mass 1.5 filter (AM-1.5) provides 100 mW cm^2^ of intensity, which is equivalent to normal sunlight. Prior to the exposure to the light, the reaction mixture was properly purged using nitrogen, ensuring the complete removal of oxygen/air. The gas evolved from the photocatalytic reaction was injected directly into the sample loop, which was controlled by a gas-sampling valve (GSV) equipped with a 5 Å molecular sieve column (GC Model Shimadzu-2014). The amount of H_2_ was analyzed by a thermal conductivity detector (TCD), and N_2_ was used as the carrier gas.

Thus, perovskite-type compounds play a significant role in photocatalysis and photoelectrocatalysis, with the possibility of tailoring their semiconductor properties. Perovskite-based semiconductors have been extensively explored with respect to various photocatalytic and PEC reactions. Considering the recent progress in perovskite-based solar cell photovoltaic (PV) materials, organo-lead halide perovskite-type materials are also attracting a great deal of interest because of their consistent and high solar-to-hydrogen (STH) efficiency [148]. More efforts are required to improve the kinetics of photocatalytic water splitting reactions, to achieve a hydrogen yield of practical relevance. Heterostructures (e.g., heterojunctions) with organo-halide perovskites as well as a number of other combinations of perovskite and metal oxide may show improved solar water splitting performance. The design, construction, and photocatalytic performance of semiconductor heterojunctions have been systematically reviewed by Wang *et al* [149]. The progress reported in this field so far indicates that forming heterojunctions affords a promising option for enhancing the photocatalytic efficiencies of photocatalytic semiconductors. Because only limited heterojunction-based compositions have been explored so far, this area is likely to witness much progress, with enhanced scientific activity, in years to come [150, 151].

### 1.7. Summary and future perspectives

Mixed oxides with perovskite-type structures have long been a fascinating group of materials for both academic and practical-application reasons. Because it is possible to play with ABO_3_-type perovskite symmetry with a very large number of cations, enormous efforts to exploit their electronic, electric, magnetic, and optical as well as catalytic properties have been reported. Even with numerous successes in applications related to electronics and oxygen permeability, however, perovskites have yet to find recognition in environmental catalysis, with few exceptions. Despite this history, the one single reason that can rejuvenate the potential of perovskite-based catalysts in environmental applications is the substantially decreasing sulfur content in fuels. Diesel sulfur content has already been lowered drastically in many parts of the world, and similar plans are under way even in countries like China and India. This may renew the possibility of using perovskite-based catalysts for vehicle exhaust applications, especially in direct diesel soot oxidation. Another interesting possibility may emerge for catalytic combustion of producer gas generated through biomass gasification. This mixture typically contains very high concentrations of carbon monoxide and hydrogen with traces of sulfur. We have already started exploring perovskites for producer gas combustion reactions, with initial results favoring for more detailed investigations, including stability studies. Photocatalytic water splitting is yet another area where perovskites, heterojunction-based, and other composite materials show good potential for application in the future. Advances in the synthesis of perovskites, particularly their successful synthesis on commercial cordierite and other supports, are some of the developments that have been successfully demonstrated and should certainly help in the discovery of more applications for this interesting group of materials in the future. Needless to say, despite hundreds of perovskite compositions explored so far, there may be even more to come. This research area is therefore poised to grow even more rapidly.
